# Prevalence of small for gestational age infants in 21 cities in China, 2014–2019

**DOI:** 10.1038/s41598-021-87127-9

**Published:** 2021-04-05

**Authors:** Hui He, Huazhang Miao, Zhijiang Liang, Ye Zhang, Wei Jiang, Zhi Deng, Jie Tang, Guocheng Liu, Xianqiong Luo

**Affiliations:** 1 Guangdong Women and Children Hospital of Guangzhou Medical University, Guangzhou, 511442 China; 2 Guangdong Women and Children Hospital, Guangzhou, 511442 China; 3 Department of Preventive Medicine, School of Public Health, Guangzhou Medical University, Guangzhou, 511436 China

**Keywords:** Diseases, Health care, Medical research, Epidemiology, Paediatric research

## Abstract

Infants who are small for gestational age (SGA) are at increased risk of neonatal and infant death, non-communicable diseases and growth retardation. However, the epidemiological characteristics of SGA remain unclear. We aim to explore the prevalence of SGA and to examine its socioeconomic associations by using data from 21 cities. 10,515,494 single live birth records between 2014 and 2019 from the Guangdong Women and Children Health Information System were included in the study. Descriptive statistical methods were used to analyze the prevalence trend of SGA and its distribution. We also analyze the associations between the prevalence of SGA and per-capita GDP. The prevalence of SGA in Guangdong Province from the years 2014–2019 was 13.17%, 12.96%, 11.96%, 12.72%, 11.45%, 11.30% respectively, and the overall prevalence was 12.28%. The prevalence of term SGA infants in Guangdong Province was 12.50%, which was much higher than that of preterm SGA (7.71%). There was a significant negative correlation between the SGA prevalence and per-capita GDP in 21 cities of Guangdong Province. The level of economic development may affect the prevalence of SGA. The prevalence of SGA in full term infants is significantly higher than in premature infants, suggesting that most SGA infants may be born at a later gestational age.

## Introduction

Small for gestational age (SGA) infants, defined as infants with a birth weight below the 10th percentile of an optimal standard reference population for the sex and gestational age in a region^1^, remains an important public health concern worldwide, although nutrition levels have improved across the last 30 years. Previous studies have suggested that SGA increases the risk of neonatal death and infant death. It also has a long-term impact on individual health outcomes, such as stunting and adult-onset metabolic risks^2,3^. The prevalence of SGA ranges between 3 and 10%^4^ with two-thirds of SGA cases occurring in Asia. The prevalence of SGA in China is about 6.5%, and the total number of SGA births is the fifth highest in the world^5^. Guangdong Province is one of the most economically prosperous and populous provinces in South China. Since the implementation of the national two-child policy, there has been a lack of the latest epidemiological data on SGA. The purpose of this study is to analyze the prevalence of SGA in 21 cities of Guangdong Province by using birth certificate data, and to shed light on the relationship between SGA and its socio-economic evolution to provide the basis for the promotion or formulation of SGA prevention, intervention measures and early-childhood development projects.

## Results

**Figure 1:**
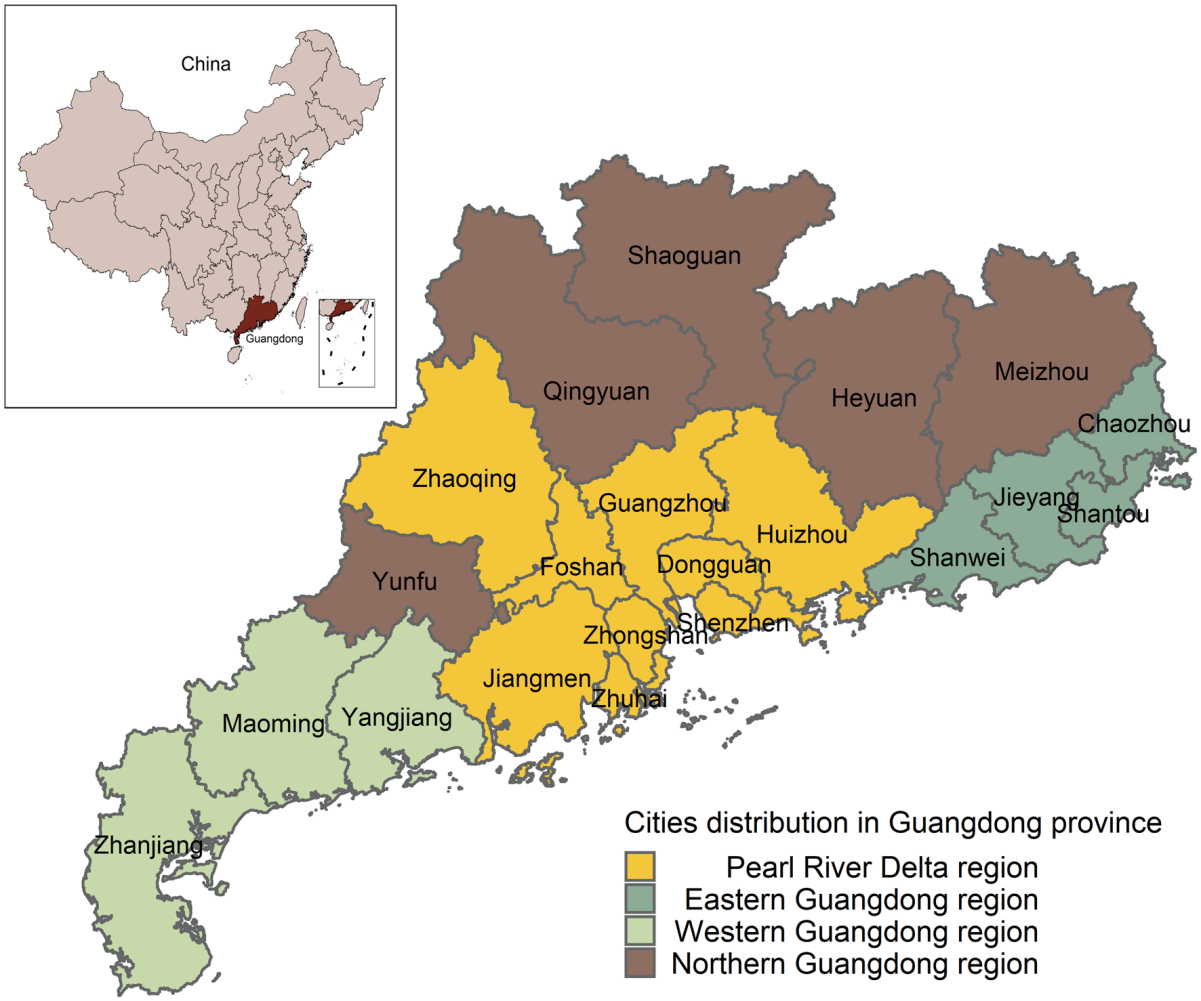
Geographical location of the study. Guangdong Province is located in southern China and is divided into four regions: the Pearl River Delta, Eastern, Western, and Northern Guangdong, which are marked in yellow, green, gray and brown, respectively.

Guangdong Province, located in southern China, ranks first among China's provincial economies and consists of 21 cities, with a resident population of about 11.521 million people^6^ (Fig. 1). During the study period, from Jan 1, 2014 to Dec 31, 2019, 10,515,494 singletons infants were born, including 5,617,151 male infants and 4,898,343 female infants. Also, there were 621,993 (48.16%) SGA newborns in the Pearl River Delta region and 669,618 (51.84%) in non-Pearl River Delta regions (Table 1).

**Table 1:** Prevalence of SGA (per 100 infants) in Guangdong province, China, 2014–2019.

Variables	Years/regions	Term	Preterm	Male	Female	Total
Number of births	2014	1,745,020	77,965	980,027	842,958	1,822,985
	2015	1,606,007	73,114	900,047	779,074	1,679,121
	2016	1,767,088	88,036	989,361	865,763	1,855,124
	2017	1,798,256	81,084	1,000,842	878,498	1,879,340
	2018	1,590,641	80,017	889,316	781,342	1,670,658
	2019	1,531,260	77,006	857,558	750,708	1,608,266
	2014–2019	10,038,272	477,222	5,617,151	4,898,343	10,515,494
Number of SGAs	2014	233,767	6229	125,681	114,315	239,996
	2015	211,787	5865	114,354	103,298	217,652
	2016	215,455	6414	115,379	106,490	221,869
	2017	232,697	6323	125,822	113,198	239,020
	2018	185,206	6066	99,564	91,708	191,272
	2019	175,926	5876	95,083	86,719	181,802
	2014–2019	1,254,838	36,773	675,883	615,728	1,291,611
Prevalence of SGAs	2014	13.40	7.99	12.82	13.56	13.17
	2015	13.19	8.02	12.71	13.26	12.96
	2016	12.19	7.29	11.66	12.30	11.96
	2017	12.94	7.80	12.57	12.89	12.72
	2018	11.64	7.58	11.20	11.74	11.45
	2019	11.49	7.63	11.09	11.55	11.30
	2014–2019	12.50	7.71	12.03	12.57	12.28
Number of births	Pearl River Delta	5,505,543	292,329	3,107,779	2,690,093	5,797,872
	Eastern	1,571,807	56,401	853,839	774,369	1,628,208
	Western	1,572,175	62,295	877,967	756,503	1,634,470
	Northern	1,388,747	66,197	777,566	677,378	1,454,944
Number of SGAs	Pearl River Delta	600,340	21,653	328,636	293,357	621,993
	Eastern	239,229	4980	125,741	118,468	244,209
	Western	229,786	5100	122,505	112,381	234,886
	Northern	185,483	5040	99,001	91,522	190,523
Prevalence of SGAs	Pearl River Delta	10.90	7.41	10.57	10.91	10.73
	Eastern	15.22	8.83	14.73	15.30	15.00
	Western	14.62	8.19	13.95	14.86	14.37
	Northern	13.36	7.61	12.73	13.51	13.09

*SGA*: small for gestational age.

### General characteristics

As depicted in Table 2, the mean maternal age of the population was 27.88 ± 5.09 years, mostly of Han ethnicity (95.7%) and urban population (68.8%). Furthermore, the ratio of maternal ages less than 20, 20–24, 25–29, 30–34, 35–39 and more than 40 was 3.5%, 22.8%, 39.3%, 23.4%, 8.9%, 2.0%, respectively. In addition, more than half of babies were born by vaginal delivery (61.1%). A pooled total of 10,505,500 singleton live births (99.9%), 9994 cases involving stillbirths, death within a few days after birth or unknown pregnancy outcome were included across the 6 specific years in the study. The average gestational age of the population was 38.84 ± 1.45 weeks, and the average birth weight was 3171.21 ± 436.43 g. Among these, there were 1,291,611 SGA newborns—1,254,838 term SGA (97.2%) and 36,773 preterm SGA (2.8%). Males accounted for 52.3% of SGA births, and females accounted for 47.7% of cases.

**Table 2:** Percentage of SGA and non-SGA across different groups stratified by characteristics of mothers and infants.

Characteristics	Non-SGA	SGA	Total	*t/x*^*2*^	*P*
**Number of births, n**	9,223,883	1,291,611	10,515,494		
**Residential areas, n (%)**					
Urban	6,413,077 (69.5)	818,624 (63.4)	7,231,701 (68.8)	19,932.26	< 0.001
Rural	2,810,806 (30.5)	472,987 (36.6)	3,283,793 (31.2)		
**Maternal age, n (%)**					
Mean ± SD	28.02 ± 5.08	26.82 ± 5.07	27.88 ± 5.09	252.98	< 0.001
< 20	298,016 (3.2)	71,807 (5.6)	369,823 (3.5)	67,808.57	< 0.001
20–24	2,024,792 (22.0)	372,922 (28.9)	2,397,714 (22.8)		
25–29	3,632,845 (39.4)	504,067 (39.0)	4,136,912 (39.3)		
30–34	2,223,863 (24.1)	237,467 (18.4)	2,461,330 (23.4)		
35–39	853,419 (9.3)	84,294 (6.5)	937,713 (8.9)		
≥ 40	185,342 (2.0)	20,129 (1.6)	205,471 (2.0)		
Un-know	5606 (0.1)	925 (0.1)	6531 (0.1)		
**Parity, n (%)**					
Nulliparous	2,927,499 (31.7)	506,352 (39.3)	3,433,851 (32.7)	51,513.41	< 0.001
Parous	4,330,525 (46.9)	470,764 (36.4)	4,801,289 (45.7)		
Un-know	1,965,859 (21.4)	314,495 (24.3)	2,280,354 (21.6)		
**Method of delivery, n (%)**					
Caesarean section	2,239,153 (24.3)	251,626 (19.5)	2,490,779 (23.7)	14,570.74	< 0.001
vaginal	5,592,653 (60.6)	827,212 (64.0)	6,419,865 (61.1)		
Un-know	1,392,077 (15.1)	212,773 (16.5)	1,604,850 (15.3)		
**Pregnancy outcomes, n (%)**					
Live birth	9,215,009 (99.9)	1,290,491 (99.9)	10,505,500 (99.9)	13.936	0.003
Stillbirth	19 (0.0)	3 (0.0)	22 (0.0)		
Dead within 7 days	147 (0.0)	27 (0.0)	174 (0.0)		
Un-know	8708 (0.1)	1090 (0.1)	9798 (0.1)		
**Gestational age, weeks, n (%)**					
Mean ± SD	38.81 ± 1.47	39.03 ± 1.29	38.84 ± 1.45	159.22	< 0.001
24–27	3196 (0.0)	56 (0.0)	3252 (0.0)	10,101.17	< 0.001
28–32	48,977 (0.5)	2854 (0.2)	51,831 (0.5)		
33–36	388,276 (4.2)	33,863 (2.6)	422,139 (4.0)		
37–42	8,783,434 (95.2)	1,254,838 (97.2)	10,038,272 (95.5)		
**Infants sex, n (%)**					
Male	4,941,268 (53.6)	675,883 (52.3)	5,617,151 (53.4)	701.994	< 0.001
Female	4,282,615 (46.4)	615,728 (47.7)	4,898,343 (46.6)		
**Birth weight, g**					
Mean ± SD	3249.7 ± 397.8	2611.0 ± 250.8	3171.21 ± 436.43	1775.75	< 0.001
< 1500	18,392 (0.2)	7058 (0.5)	25,450 (0.2)	3811.67	< 0.001
1500–1999	42,185 (0.5)	24,389 (1.9)	66,574 (0.6)		
2000–2499	140,194 (1.5)	218,471 (16.9)	358,665 (3.4)		
2500–2999	1,651,555 (17.9)	1,024,705 (79.3)	2,676,262 (25.5)		
3000–3499	4,968,695 (53.9)	16,988 (1.3)	4,985,683 (47.4)		
3500–3999	2,069,499 (22.4)	0 (0.0)	2,069,499 (19.7)		
≥ 4000	333,363 (3.6)	0 (0.0)	333,363 (3.2)		

### Prevalence of SGA

**Figure 2:**
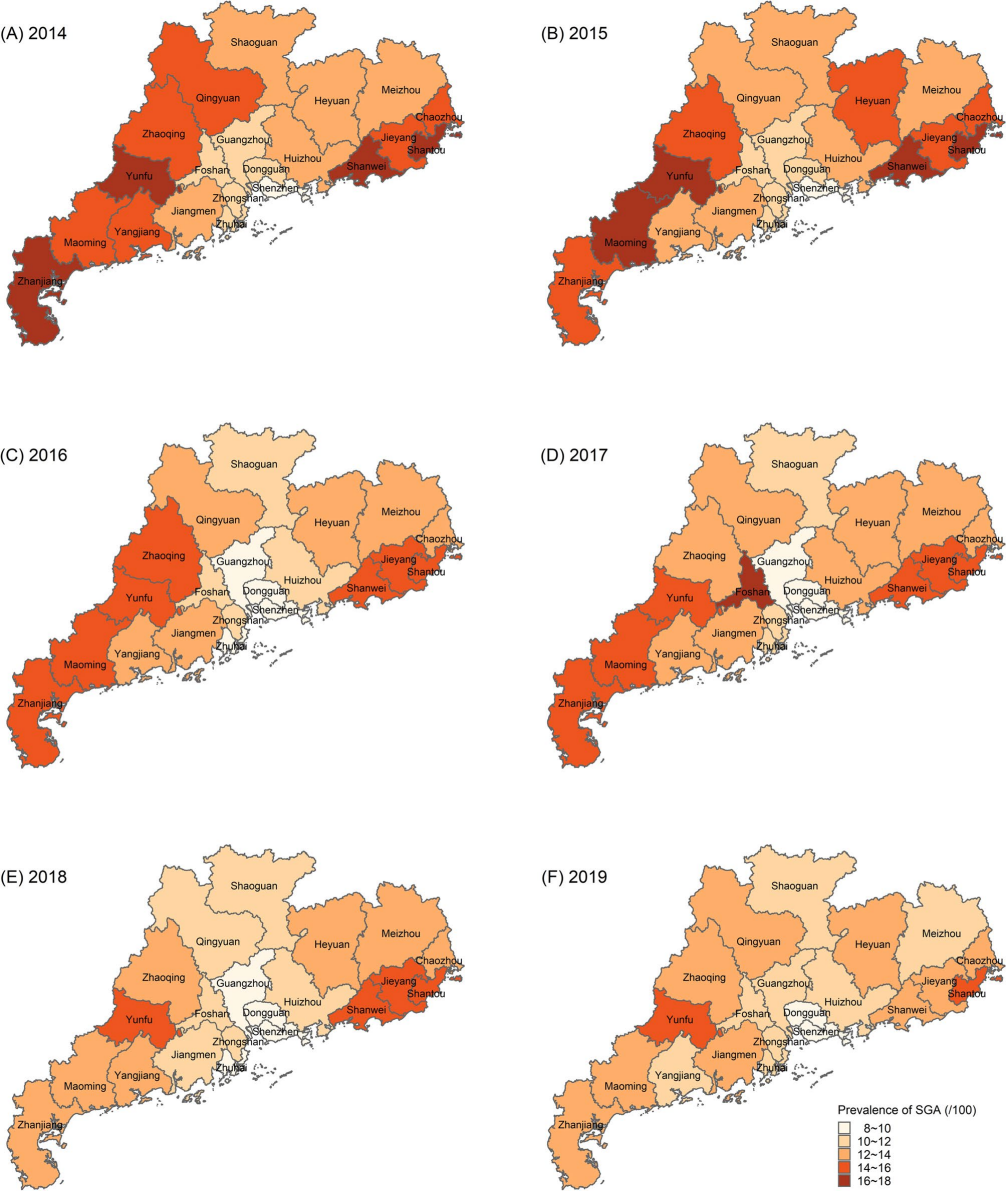
Regional Distribution of SGA prevalence in Guangdong Province, China from 2014 to 2019. (**A**) Regional distribution of SGA prevalence in 2014. (**B**) Regional distribution of SGA prevalence in 2015. (**C**) Regional distribution of SGA prevalence in 2016. (**D**) Regional distribution of SGA prevalence in 2017. (**E**) Regional distribution of SGA prevalence in 2018. (**F**) Regional distribution of SGA prevalence in 2019.

From 2014 to 2019, the overall prevalence of SGA in 21 cities was about 12.28%, ranging from 11.30 up to 13.17% (Table 1). Although the prevalence of SGA increased in 2017, primarily in the Pearl River Delta region, it showed a slow downward trend as a whole (Fig. 2).

**Figure 3:**
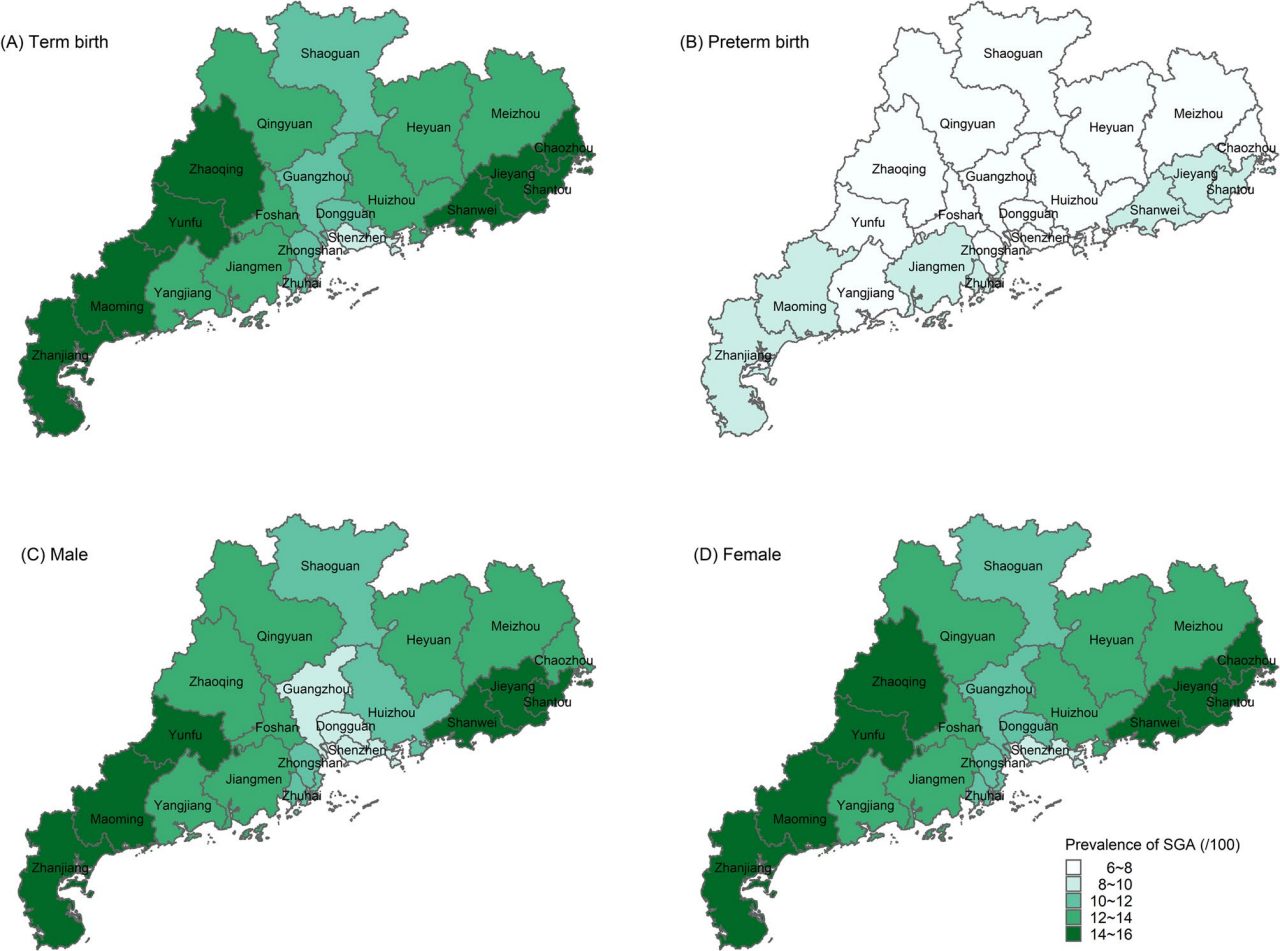
Different category of SGA prevalence in Guangdong Province, China. (**A**) Regional distribution of SGA prevalence for term infants. (**B**) Regional distribution of SGA prevalence for preterm infants. (**C**) Regional distribution of SGA prevalence for male infants. (**D**) Regional distribution of SGA prevalence for female infants.

Table 1 illustrated the prevalence and number of SGA for term, preterm, male, female according region and years. The prevalence of SGA was 12.03% in boys and 12.57% in girls, and the difference was statistically significant (*p* < 0.05). During the study, the prevalence of SGA in girls was higher than that in boys. Although a downward trend was observed among both sexes, the decline of SGA in female infants was more obvious (Supplementary Figs. S1 and S2). In 21 cities, the overall prevalence of term SGA was 12.50%, and prevalence of preterm SGA was 7.71%. Again, this difference was statistically significant (*p* < 0.05). In the composite population, preterm SGA accounted for 0.35% and term SGA accounted for 11.93%; the ratio of term SGA to preterm SGA was 34.12 (Fig. 3).

### Regional economic characteristics of Guangdong Province

From 2014 to 2019, the per-capita GDP of Guangdong Province in CNY is 63,452, 67,503, 72,787, 81,089, 86,412, 94,172. In addition, the per capita GDP of the Pearl River Delta region is CNY 116,440. However, it is only CNY 35,320 in the economically underdeveloped non-Pearl River Delta region. According to the Statistical Bulletin of Guangdong National Economic and Social Development, the GDP of the core areas of the Pearl River Delta will account for about 80.7% of the province's GDP in 2019, and the GDP of the eastern, western and northern regions of Guangdong will account for 6.4%, 7.1% and 5.8% respectively. From these figures, it is evident that regional development is extremely unbalanced, with the development of the non-Pearl River Delta region far behind that of the Pearl River Delta.

### Prevalence of SGA in 21 cities

From 2014 to 2019, the overall prevalence of SGA in the Pearl River Delta region is around 10.74%, ranging between 9.96 and 11.86%. For the same period, the prevalence in the non-Pearl River Delta region is approximately 14.33%, which is substantially higher than that in the Pearl River Delta region, and reaches statistical significance (*p* < 0.05). The prevalence of SGA ranges between 13.75% and 16.05% in eastern Guangdong, 12.93–15.80% in western Guangdong, and 12.20–14.06% in northern Guangdong. The prevalence of SGA among different cities in the non-Pearl River Delta area is unbalanced, especially in eastern and western Guangdong. At the same time, we found that the prevalence of SGA in the non-Pearl River Delta region never fell below 10%, while that in Shenzhen never exceeded 10%. Even so, prevalence varied in the remaining cities, indicating that SGA was more concentrated in regions with poor socioeconomic status (Table 1). With the regional development of economy, the gap between SGA prevalence in the Pearl River Delta and non-Pearl River Delta regions gradually narrowed, regardless of term or preterm (Supplementary Figs. S3 and S4).

### Correlation analysis between SGA and per-capita GDP

**Figure 4:**
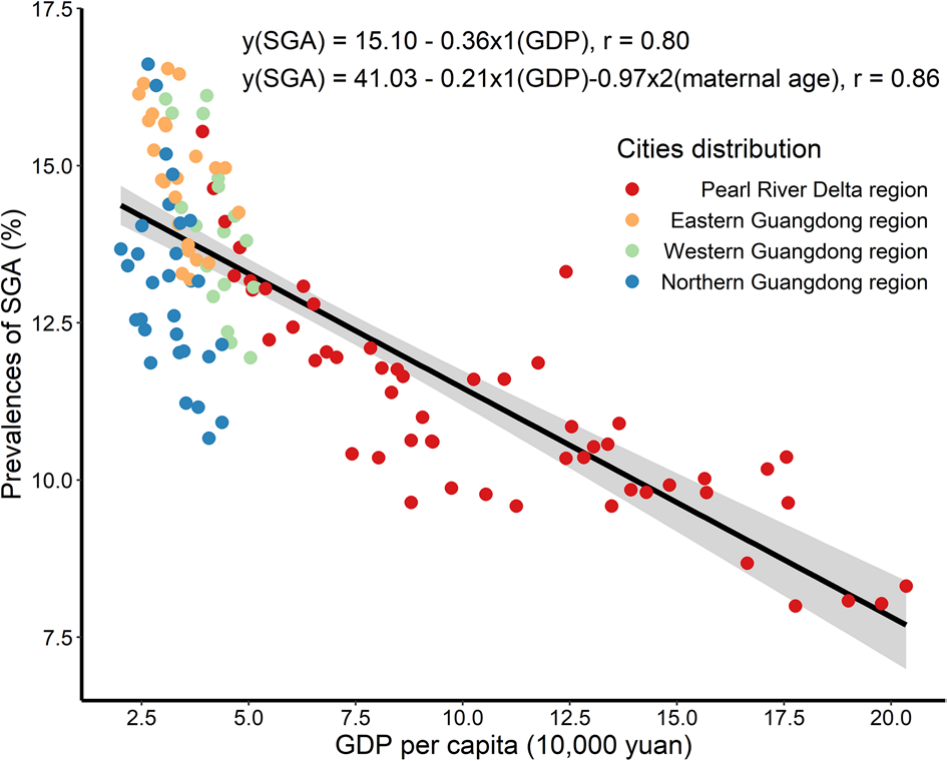
The correlation between SGA and per-capita GDP, China from 2014 to 2019. The Pearl River Delta, Eastern, Western, and Northern Guangdong, which are marked in red, orange, green, blue, respectively.

Figure 4 shows the per-capita GDP and SGA prevalence of 21 cities in Guangdong Province. The correlation analysis shows that SGA has a significant negative correlation with GDP per capita (*r* = 0.80, *p* < 0.01), and has a good linear relationship. Furthermore, we have used the confounding factors of maternal ages to do a multivariate analysis. Finally, we have found that the negative correlation between SGA and per-capita GDP, maternal ages (*r* = 0.86, *p* < 0.01). With the growth of maternal age, personal income and savings increased, which also signified a rise in economic level for family. Therefore, the more developed the economy, the lower the prevalence of SGA.

## Discussion

This study demonstrates the prevalence of SGA from 2014 to 2019 and the correlation between SGA prevalence and GDP per capita in 21 cities of Guangdong Province. These findings help further explore the causes of geographical differences in SGA prevalence. Moreover, a major strength of our study is that it is based on overall population data with a large sample size. In the early stage, using the household registration location information to find study subjects for inclusion, we conducted a spatiotemporal analysis of adverse birth outcomes in Guangdong Province from 2014 to 2017, and found that the prevalence of SGA was about 12.86%^7^. However, we focused on babies born and reported in 21 cities of Guangdong, regardless of the location of household registration. In view of the population mobility and richer population, its epidemic trend is more representative of Southern Han Chinese.

### Prevalence

More than 30 million SGA infants are born each year worldwide. A recent population-based study of live births of infants in Sweden found a 5.7% prevalence of moderate SGA (birth weights at or above the 3rd percentile and less than the 10th percentile) and a 2.1% prevalence of severe SGA (birth weights below the 3rd percentile)^8^. According to the WHO’s Multi-Country Survey on Maternal and Newborn Health, the prevalence of SGA was relatively high in Cambodia (18.8%), Nepal (17.9%), and Japan (16.0%), and indicated that 35.1% of SGA prevalence worldwide may be related to short stature of the mother^9,10^. Mikolajczyk et al.^11^ systematically studied 237,025 maternal and birth data records, and concluded that maternal height and weight are determinants of fetal weight. It is possible that ethnicity is the variable most closely correlated with fetal weight. Buck Louis, et al.^12^ recruited 2334 women comprised of non-Hispanic whites, non-Hispanic blacks, Hispanics, and Asians in the United States. They found that estimated fetal weight (EFW) differed significantly by ethnicity > 20 weeks of gestation. That is to say that incorporating ethnic-specific criteria could improve the accuracy of fetal growth assessment. In our study, the prevalence of SGA was about 12.28%, which was far from the 6.61% reported in the survey data from the Chinese Pediatric Society in 2005. However, our findings were based on ethnicity and the adoption of a new birth-weight reference suitable for China’s background. Moreover, the reported prevalence in this study is close to that of other Asian countries. At the same time, the SGA prevalence in 21 cities of Guangdong Province, a microeconomic province in China, has certain applicability to a broader population.

It is worth mentioning that previous studies have shown that being male is a risk factor for preterm birth^13,14^. Nevertheless, we find that SGA infants, who also have a low birth weight, are more commonly female infants under the sex- and gestational age-specific reference. In the United States, an epidemiological study of term SGA cases likewise showed that females had a higher prevalence of SGA than males^15^. When a recent study using data from rural Nepal and southern India compared the prevalence and mortality of SGA associated with using different birthweight references, the authors also concluded that there was a higher prevalence of SGA among females when using reference populations that were not sex-specific. Otherwise, there was no significant difference in the prevalence of SGA^16^. Our study results were slightly different. The reasons for those differences may be related to the sample size and inclusion criteria, and merits further analysis of the association between gender and SGA.

A meta-analysis of mortality risk in preterm and SGA infants from low- and middle-income countries was published in 2010, and found that SGA cases were more common in term infants than in premature infants in Africa and Asia^17^. In addition, among a dataset of 19,269 newborns from Tanzania, Africa, 15.8% were term SGA, and 0.3% preterm SGA^18^. In 2017, a population-based retrospective cohort study investigated 248,501 couples and their children in China. The results showed that there were 11,474 premature singleton live births, which included 317 preterm SGA infants (2.77%). Compared to North China, the prevalence of preterm SGA was higher in South China, which may be related to the significant differences in height and weight between Northern and Southern Han Chinese^19^. The above studies are consistent with the results of our study, which may indicate that SGA infants are born at a later gestational age in Asians and Africans. A study from Canada, which included 23,788 singleton preterm infants aged 24–28 weeks through the International Network for Evaluating Outcomes in Neonates (iNeo), also found that SGA occurred closer to later gestational ages than non-SGA cases. At the same time, preterm SGA infants had a higher mortality rate when gestational age < 29 weeks^20^. Preterm SGA confers a higher risk of neonatal mortality risk beyond premature birth alone, since preterm SGA involves two factors: preterm and SGA. A retrospective study found that among premature infants with severe SGA (birth weight below the fifth percentile of the sex- and gestational age-specific reference), the mortality rate was 6%, while the mortality rate of infants without severe SGA was 2.29%^21^. Although preterm SGA accounts for a low proportion of the population, we need to pay more attention to its prevalence and prognosis and individualize the management of such cases with the goal of reducing neonatal mortality.

### Economic characters and prevalence of SGA

Since its reform and opening, Guangdong Province has flourished economically by virtue of its numerous geographic advantages (adjacency to Hong Kong, Macao and overseas Chinese), as well as national policy advantages. Since then, it has risen to become the most prosperous and developed province in southern China; however, there are still non-negligible differences in economic development, medical security level and per-capita education across its regions. Urban distribution is predominant in the Pearl River Delta region, while villages and towns are dominant in eastern, western, and northern Guangdong. In recent years, the development gap between the non-Pearl River Delta region and the Pearl River Delta region has gradually expanded; the Pearl River Delta region is currently densely populated, and has rich medical resources and economic strengths. Due to the liberalization of the comprehensive two-child policy in 2016, SGA births have also been increasing to a certain extent. There is a small peak in prevalence in 2017, which tells us that the fluctuation of the prevalence of SGA was impacted by the introduction of China's population policy.

Some studies have reported that maternal socioeconomic status has an important impact on the prevalence of SGA. Dividing per capita income into five intervals, one study found that the prevalence of SGA was 8.9% for infants from households in the lowest quintile of income compared with 5.6% of infants in the highest quintile^22^. Furthermore, in a Finnish study, people with low socioeconomic status were found to have an 11–24% higher risk of developing SGA than those with high socioeconomic status^23^. Shenzhen is an economically developed area in Guangdong province, and its SGA prevalence never exceeded 10% during the period studied. Our study also further evaluated the relationship between SGA prevalence and economic development level using Pearson's correlation coefficient, which showed a significant negative correlation between SGA incidence and GDP per capita. In addition, according to data from the mid- to late twentieth century in the United States, the prevalence of SGA in economically underdeveloped areas was 19.7%; however, with the development of the economy, the SGA rate could be reduced to 13.7%, showing a straight downward trend^24^. Similarly, in order to promote the coordinated development of a regional economy, Guangdong Province issued a series of policy guidelines to promote the revitalization and development of mountainous areas in eastern, western and northern Guangdong, which improved the coordination of sectional development. From the analysis of the results, the prevalence of SGA varied significantly among the 21 cities. With regard to the epidemiological trend, the prevalence of SGA in each area showed a slow decline, which was closely related to the area’s regional economic characteristics and evolution.

With regard to the above outcomes, it may be that in economically developed areas, pregnant women have a higher level of education, are not required to undertake as much physical work during pregnancy, have a higher material living standard, and pay greater attention to maternal and child health care and nutritional supplementation. Earlier studies have also shown that the prevalence of low birth weight infants is influenced by factors such as maternal occupation and level of education^25^. Secondly, in areas with lower per-capita income, pregnant mothers are mostly of short stature^22^. The relatively unsound medical facilities in economically underdeveloped areas may also increase the risk of SGA.

In this study, by analyzing the prevalence and economic evolution characteristics of SGA in 21 cities, we found an imbalance in the prevalence of SGA between regions and explored the possible influence of GDP per capita on the prevalence of SGA. Our findings not only provide a basis for a nationwide multicenter, large-sample neonatal survey, but can also pave the way for subsequent prevention and control of SGA.

There are some limitations to our study. Factors such as individual family economic differences, maternal health disease status, age and education level, and humanistic customs in each region were not included in the discussion, and need to be further analyzed in subsequent studies.

## Materials and methods

### Data sources and data collection

We used data from the Guangdong Women and Children Health Information System to conduct this analysis. This system documents information on all births, which includes place of birth, date of birth, gender, gestational week, weight, length, mother's age, etc. All these data are filled in system by the hospital staff or midwives according to the babies’ medical records, which are also checked by obstetricians. This study collected birth information on singleton births at 24–42 weeks. GDP per capita was obtained from the Guangdong Statistical Yearbook between 2014 and 2019 and the Statistical Bulletin of Guangdong National Economic and Social Development in 2019 (Data sources: http://stats.gd.gov.cn/).

### Diagnostic criteria for SGA

SGA means that the birth weight is below the 10th percentile based on the sex- and gestational age-specific references. We adopt the latest weight standard commonly used in clinical practice in China, which is in line with the domestic background. That is, the Chinese neonatal birth weight curve for different gestational ages serves as the diagnostic basis^26^. We classified term SGA as infants born with SGA at 37–42 weeks of gestation, with preterm SGA defined as delivery of an SGA infant prior to 37 weeks of gestation.

### Regional division

In this study, the 21 cities in Guangdong Province were divided into regions according to their geographical location and economic conditions: the Pearl River Delta, Eastern Guangdong, Western Guangdong and Northern Guangdong. The Pearl River Delta region includes Guangzhou, Shenzhen, Foshan, Dongguan, Zhuhai, Zhongshan, Jiangmen, Zhaoqing and Huizhou; Eastern Guangdong includes Chaozhou, Shantou, Shanwei and Jieyang; Western Guangdong includes Zhanjiang, Maoming and Yangjiang; and Northern Guangdong includes Shaoguan, Heyuan, Meizhou, Qingyuan and Yunfu (Data sources: http://www.gd.gov.cn/).

### Statistical analysis

All data were analyzed using R software (version 4.0.2), and maps were plotted using R packages (including ggplot2, jsonlite, mapproj, and Cairo). Mean and standard deviation were used to describe continuous data, and frequencies and percentages were used to describe categorical data. The comparison of rates was performed by *x*^2^ test, and the correlation analysis was conducted via Pearson's test. The difference was considered to be statistically significant if *p* < 0.05.

### Ethics approval

This study was approved by the ethics committee of Guangdong Women and Children Hospital (Approved no. 202001193).

### Informed consent

We had confirmed that all methods were performed in accordance with the relevant guidelines and regulations. The need for informed consent was waived by the ethics committee review board of Guangdong Women and Children Hospital.

## Conclusion

The prevalence of SGA showed a slow downward trend in 21 cities alongside advances in the economy and people's living standards. The level of economic development in a region may affect the prevalence of SGA in that region. The prevalence of SGA in term infants is significantly higher than that in premature ones, suggesting that SGA infants may be mostly born at a later gestational age.

## Supplementary Information

Supplementary Information

## Data Availability

No additional data are available.
