# Supplementary material for: Prevalence of small for gestational age infants in 21 cities in China, 2014–2019
Source: Sci Rep. 2021 Apr 5;11:7500. doi: 10.1038/s41598-021-87127-9 (PMC8021546; doi:10.1038/s41598-021-87127-9)
Supplement: Supplementary file 1 — Supplementary Information [file 41598_2021_87127_MOESM1_ESM.docx]

**Prevalence of small for gestational age infants in 21 cities in China, 2014-2019**

Hui He **^1^**^,^ **^†^**, Huazhang Miao **^2^**^,^**^†^**, Zhijiang Liang ^2^, Ye Zhang ^2^, Wei Jiang ^2^, Zhi Deng ^2^, Jie Tang ^3^, Guocheng Liu ^2, *^, Xianqiong Luo ^2, *^

**^1^**Guangdong Women and Children Hospital of Guangzhou Medical University, Guangzhou, 511442, China

**^2^** Guangdong Women and Children Hospital, Guangzhou, 511442, China

**^3^** Department of Preventive Medicine, School of Public Health, Guangzhou Medical University, Guangzhou, 511436, China

**^†^** These authors contributed equally, and they are the co-first authors in this study.

**^*^Corresponding author:**

Guocheng Liu, Guangdong Women and Children Hospital, Guangzhou, 511442, China (Email: hnliuguocheng@126.com)

Xianqiong Luo, Guangdong Women and Children Hospital, Guangzhou, 511442, China (Email: luoxqgz@126.com)


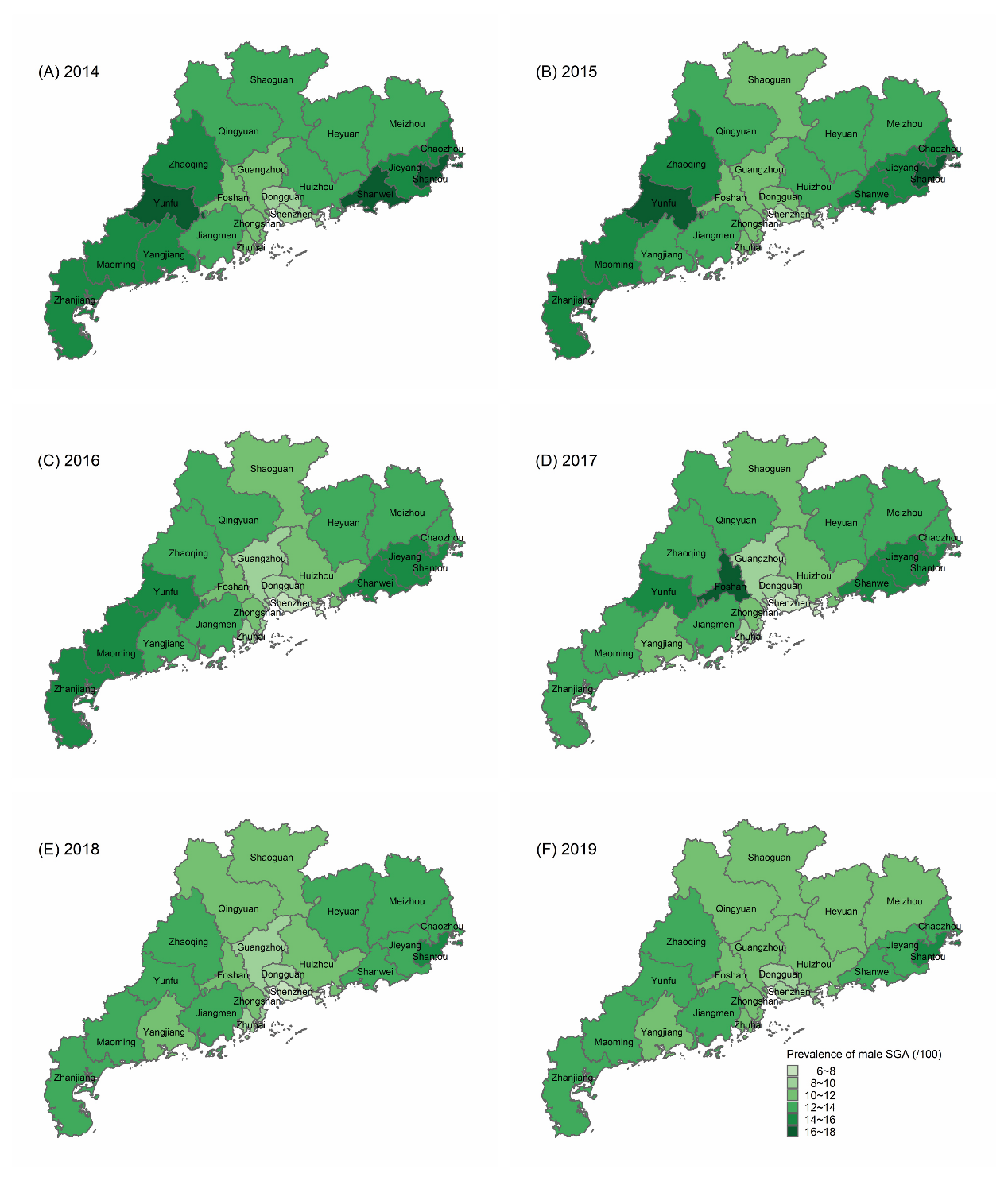


**Supplementary Figure 1**. Regional distribution of SGA prevalence for male infants in Guangdong Province, China from 2014 to 2019. (A) Regional distribution of SGA prevalence for male infants in 2014. (B) Regional distribution of SGA prevalence for male infants in 2015. (C) Regional distribution of SGA prevalence for male infants in 2016. (D) Regional distribution of SGA prevalence for male infants in 2017. (E) Regional distribution of SGA prevalence for male infants in 2018. (F) Regional distribution of SGA prevalence for male infants in 2019.


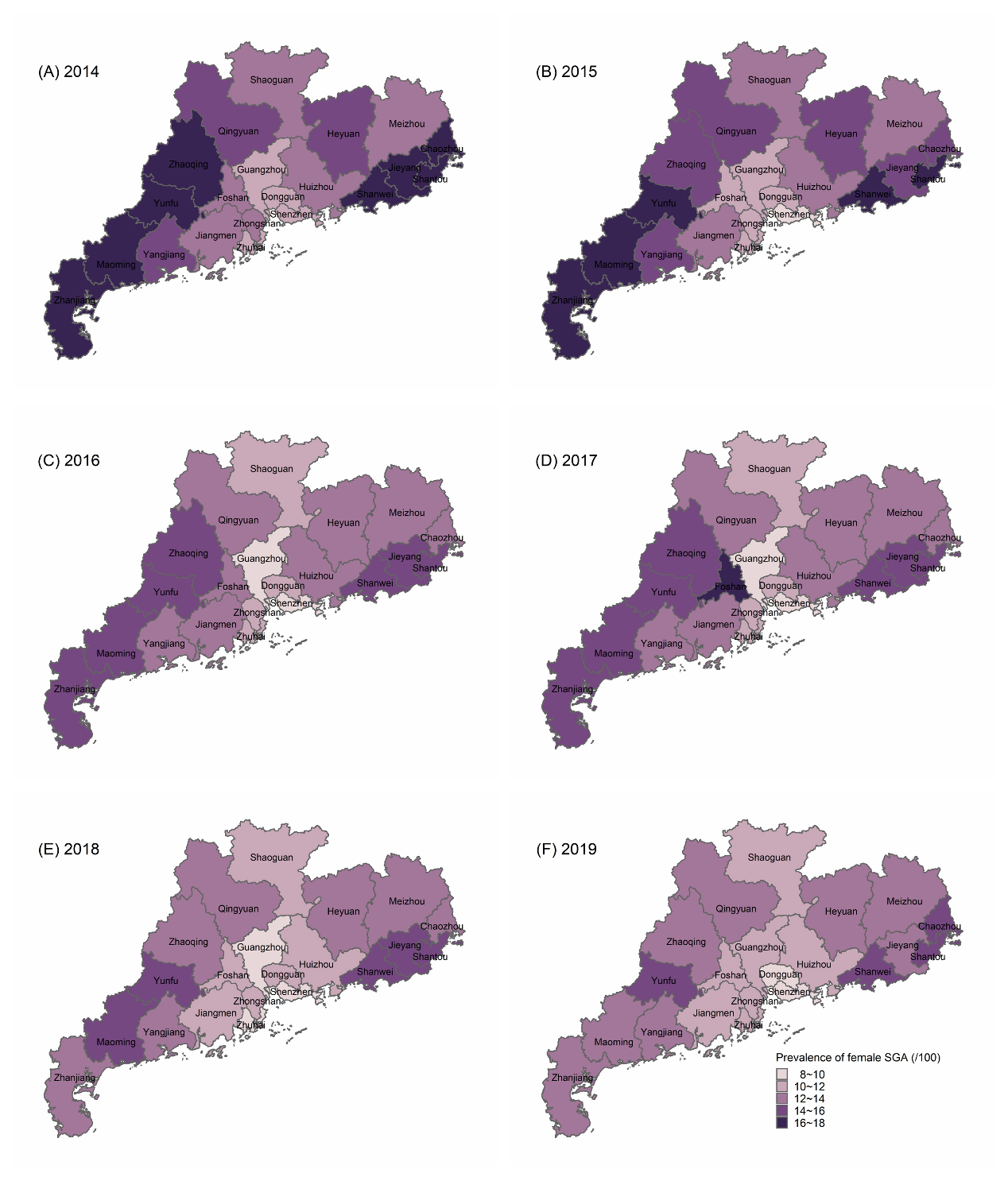


**Supplementary Figure 2**. Regional distribution of SGA prevalence for female infants in Guangdong Province, China from 2014 to 2019. (A) Regional distribution of SGA prevalence for female infants in 2014. (B) Regional distribution of SGA prevalence for female infants in 2015. (C) Regional distribution of SGA prevalence for female infants in 2016. (D) Regional distribution of SGA prevalence for female infants in 2017. (E) Regional distribution of SGA prevalence for female infants in 2018. (F) Regional distribution of SGA prevalence for female infants in 2019.


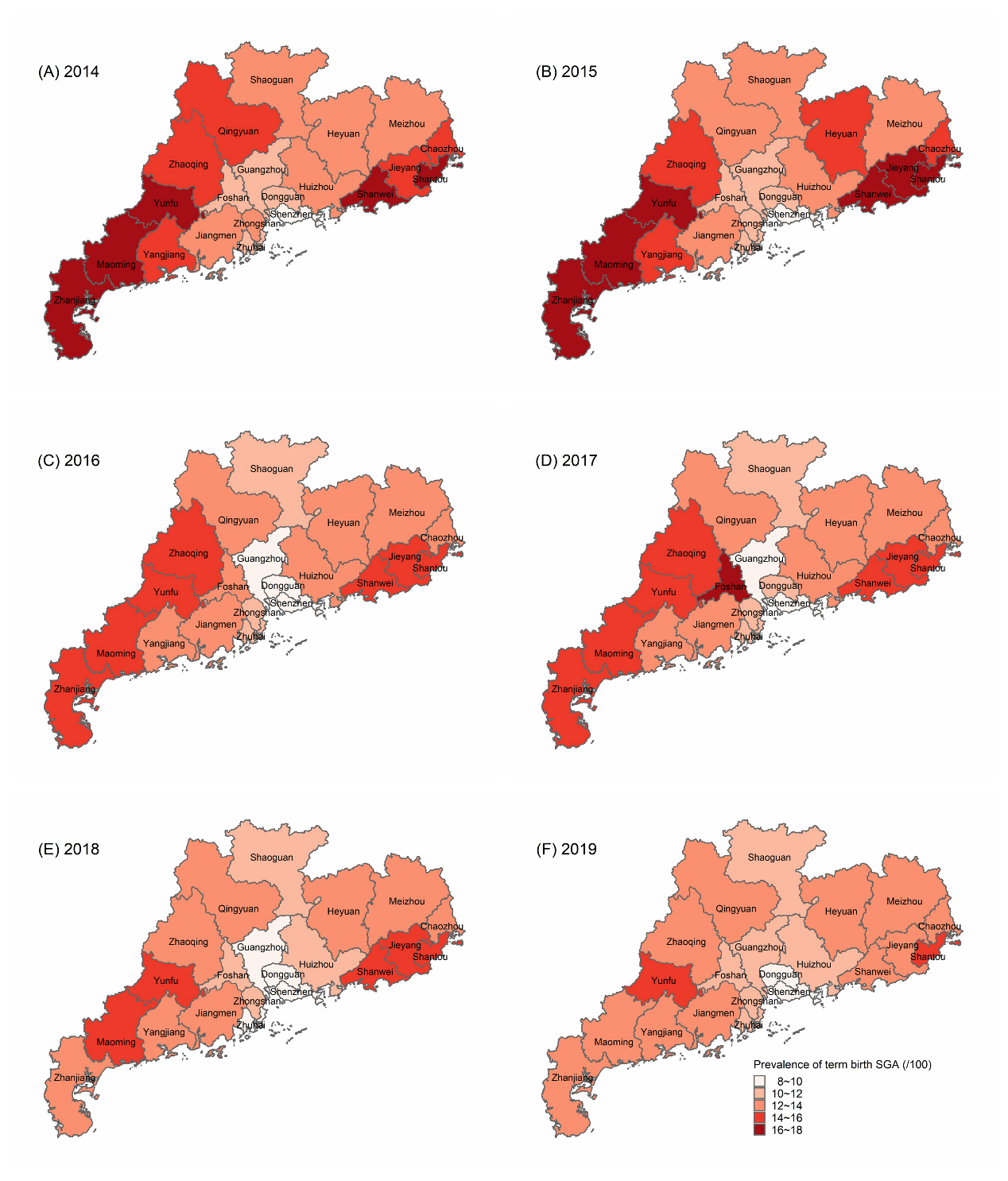


**Supplementary** **Figure 3**. Regional distribution of SGA prevalence for term infants in Guangdong Province, China from 2014 to 2019. (A) Regional distribution of SGA prevalence for term infants in 2014. (B) Regional distribution of SGA prevalence for term infants in 2015. (C) Regional distribution of SGA prevalence for term infants in 2016. (D) Regional distribution of SGA prevalence for term infants in 2017. (E) Regional distribution of SGA prevalence for term infants in 2018. (F) Regional distribution of SGA prevalence for term infants in 2019.


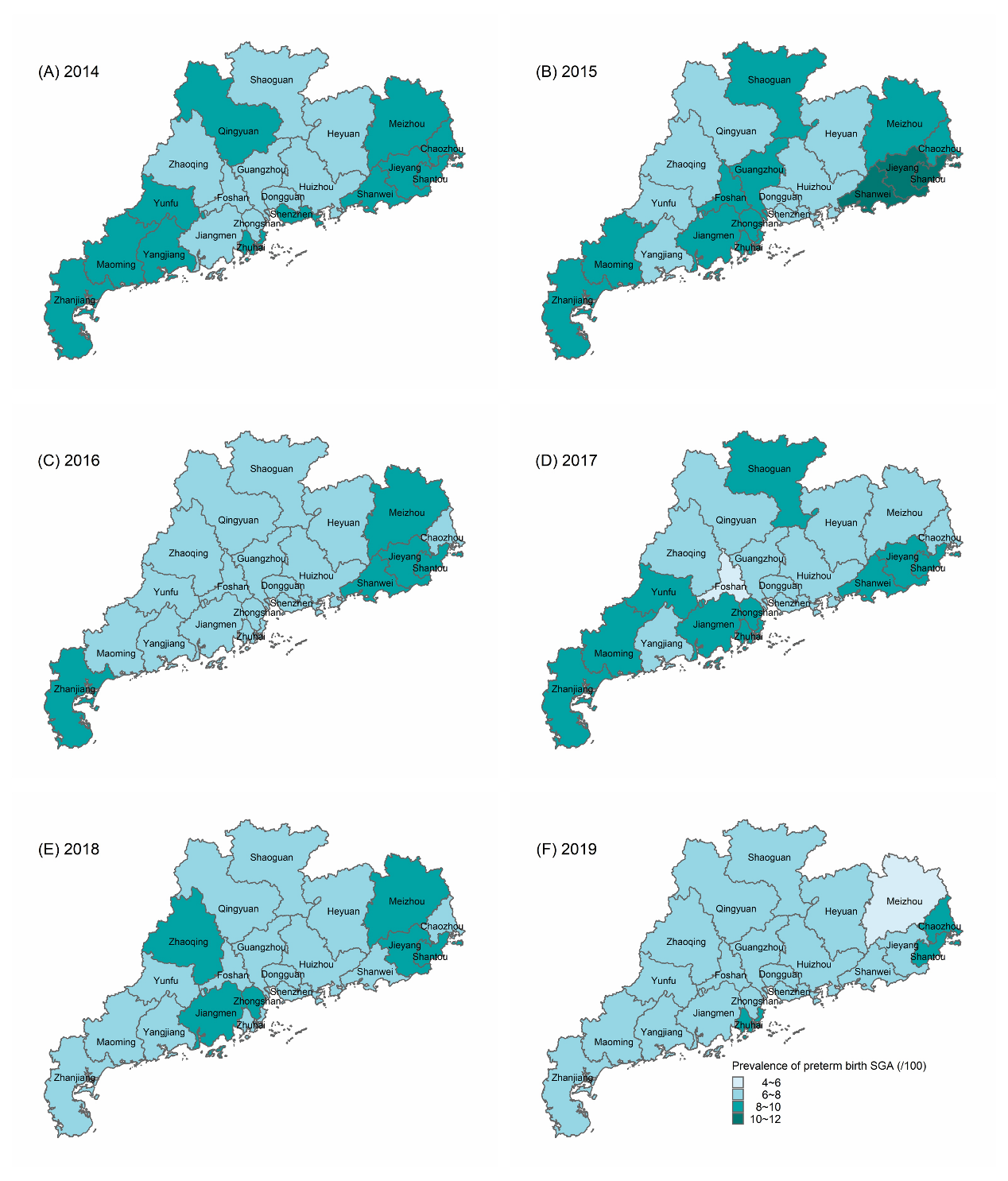


**Supplementary Figure 4**. Regional distribution of SGA prevalence for preterm infants in Guangdong Province, China from 2014 to 2019. (A) Regional distribution of SGA prevalence for preterm infants in 2014. (B) Regional distribution of SGA prevalence for preterm infants in 2015. (C) Regional distribution of SGA prevalence for preterm infants in 2016. (D) Regional distribution of SGA prevalence for preterm infants in 2017. (E) Regional distribution of SGA prevalence for preterm infants in 2018. (F) Regional distribution of SGA prevalence for preterm infants in 2019.
